# Ire1 Mediated mRNA Splicing in a C-Terminus Deletion Mutant of *Drosophila* Xbp1

**DOI:** 10.1371/journal.pone.0105588

**Published:** 2014-08-19

**Authors:** Dina S. Coelho, Catarina J. Gaspar, Pedro M. Domingos

**Affiliations:** Instituto de Tecnologia Química e Biológica, Universidade Nova de Lisboa, Oeiras, Portugal; University of Hong Kong, Hong Kong

## Abstract

The Unfolded Protein Response is a homeostatic mechanism that permits eukaryotic cells to cope with Endoplasmic Reticulum (ER) stress caused by excessive accumulation of misfolded proteins in the ER lumen. The more conserved branch of the UPR relies on an ER transmembrane enzyme, Ire1, which, upon ER stress, promotes the unconventional splicing of a small intron from the mRNA encoding the transcription factor Xbp1. In mammals, two specific regions (the hydrophobic region 2 - HR2 - and the C-terminal translational pausing site) present in the Xbp1^unspliced^ protein mediate the recruitment of the Xbp1 mRNA-ribosome-nascent chain complex to the ER membrane, so that Xbp1 mRNA can be spliced by Ire1. Here, we generated a *Drosophila* Xbp1 deletion mutant (*Excision101*) lacking both HR2 and C-terminal region, but not the Ire1 splicing site. We show that Ire1-dependent splicing of Xbp1 mRNA is reduced, but not abolished in *Excision101*. Our results suggest the existence of additional mechanisms for ER membrane targeting of Xbp1 mRNA that are independent of the C-terminal domain of *Drosophila* Xbp1^unspliced^.

## Introduction

The endoplasmic reticulum (ER) is a membranous organelle where proteins targeted for secretion or for the plasma membrane are folded and processed. Physiological conditions that impose large amounts of proteins in the ER, as for example, the production of insulin by pancreatic β-cells, may represent a challenge to the ER folding capacity [1]. The Unfolded Protein Response (UPR) is a homeostatic mechanism that attempts to balance the load of incoming proteins into the ER to its folding capacity, to avoid the accumulation of toxic misfolded proteins, which otherwise would cause ER stress [2], [3].

In higher eukaryotes, the UPR has three signaling branches triggered by different ER transmembrane proteins: protein kinase (PKR)-like ER kinase (Perk), activating transcription factor 6 (Atf6) and Inositol-requiring enzyme 1 (Ire1). Ire1 is conserved across all eukaryotes, presenting a luminal domain that detects the accumulation of misfolded proteins in the ER lumen, and a cytoplasmic domain with kinase and RNase activities that trigger downstream signaling [4], [5], [6], [7], [8]. During ER stress, Ire1 is activated and catalyzes the unconventional splicing of an intron from X-box binding protein 1 (Xbp1) mRNA (in vertebrates, *C. elegans* and *Drosophila*) or from its functional yeast (*S. cerevisiae*) homolog Hac1 [9], [10], [11], [12], [13], [14], [15]. This Ire1 mediated unconventional splicing event causes a frameshift during the translation of Xbp1 mRNA that introduces a new C-terminus with a potent trans-activation domain, generating an effective transcription factor [11], [13]. Xbp1^spliced^ enhances the expression of genes encoding ER chaperones, enzymes, and the ER protein degradation machinery [16], [17], [18]. Xbp1^unspliced^ mRNA is translated into an unstable antagonist of Xbp1^spliced^ and ATF6 signaling [19].

In addition to splicing of Xbp1 mRNA, Ire1 also cleaves a variety of mRNAs, mostly encoding proteins with signal peptide/transmembrane domains that would represent an additional challenge to the ER folding machinery under ER stress [20]. This mechanism was named RIDD (Regulated Ire1 Dependent Decay) and was also described in mammalian cells and in the fission yeast *S. pombe* (which lacks any Hac1/Xbp1 homologue) [21], [22], [23]. RIDD seems to be particularly important in cells undergoing very strong ER stress [24], [25].

The mechanism of targeting of a specific mRNA to RIDD seems to rely mostly on the existence of a signal peptide in its respective protein; the deletion of the signal peptide from known RIDD targets prevents their degradation and conversely, addition of a signal peptide to GFP is sufficient to promote the degradation of its mRNA by RIDD [26], [27]. One interesting exception is the mRNA encoding Smt3, a homologue of SUMO (small ubiquitin-like modifier), which is cleaved by RIDD although it does not have a signal peptide in its sequence [28].

Xbp1 also does not have a signal peptide in its sequence and the mechanism of recruitment of the Xbp1 mRNA to the ER membrane (and Ire1) is still unclear. Moreover, it seems that the mechanisms of recruitment to the ER membrane of Xbp1 mRNA in mammals and Hac1 mRNA in yeast are quite different. In yeast cells with no ER stress, the mRNA of unspliced Hac1 is found mostly in the cytoplasm, in association with stalled ribosomes. Upon ER stress, Hac1 mRNA is recruited to Ire1 clusters in the ER membrane, in a process that depends on a bipartite element (BE) present at the 3′ untranslated region of the Hac1 mRNA [29].

**Figure 1:**
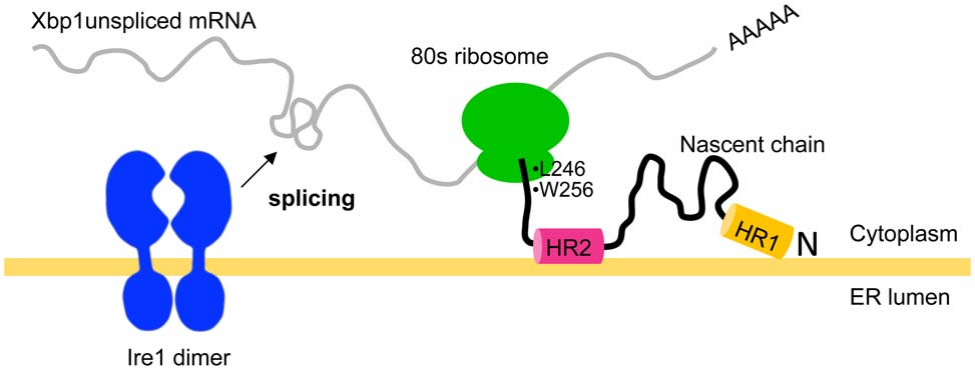
Recruitment of Xbp1 mRNA to the ER membrane in mammalian cells. XBP1^unspliced^ originates translational pausing through its C-terminal region. The hydrophobic regions (HR1 and HR2) in XBP1^unspliced^ target the XBP1 unspliced mRNA/ribosome/nascent chain complex to the ER membrane, giving the opportunity for Ire1 to splice Xbp1 mRNA. Adapted from [31].

In contrast, ER membrane localization of mammalian Xbp1 is independent of the 3′ untranslated region of Xbp1 [30]. Instead, the mRNA of Xbp1^unspliced^ is translated under normal conditions and originates a polypeptide that associates with the membrane of the ER through two hydrophobic regions (the N terminal hydrophobic region 1–HR1 and the C-terminal hydrophobic region 2–HR2) [30], [31] (Figure 1). The HR2 is a conserved region predicted to form a α-helix that has the propensity to interact with the lipid membrane [30]. Presumably, upon translation of Xbp1^unspliced^, the HR2 on the nascent polypeptide associates with the ER membrane and brings the Xbp1 mRNA-ribosome-nascent chain complex to the vicinity of Ire1, facilitating Ire1-mediated splicing. Xbp1^spliced^ lacks HR2 due to the frameshift that occurs upon Ire1-mediated splicing event and localizes predominantly in the nucleus, where it is active as transcription factor.

In addition to HR1 and HR2, there is another motif in Xbp1^unspliced^ that is also important for proper localization of Xbp1 mRNA at the ER membrane. The C-terminal region (CTR) of Xbp1^unspliced^ is essential for translational pausing, just when HR2 is protruding from the ribosome exit tunnel [31] (Figure 1). Presumably, pausing of translation stabilizes the Xbp1 mRNA-ribosome-nascent chain complex in the vicinity of the ER membrane, giving the opportunity for activated Ire1 to cleave Xbp1 mRNA [31].

In *Drosophila*, a Xbp1-EGFP ER stress reporter lacking the HR2 and CTR of Xbp1^unspliced^ was found to be activated under a variety of ER stress stimuli, including some specific physiological conditions during development, by the addition of ER stress-inducing drugs to tissues and cells or by using mutations that cause the accumulation of misfolded proteins in the ER [14], [32], [33], [34], [35], [36]. In these studies Xbp1-EGFP was expressed with the UAS/GAL4 system [37], but GFP is only observed upon the Ire1-dependent splicing of the Xbp1 intron present in the reporter. A modified “high gain” version of Xbp1-EGFP, where HR2, CTR and the 3′ UTR of Xbp1^unspliced^ were included in the reporter, greatly increased the reporter sensitivity and GFP expression upon ER stress [38]. However, in all these cases, and due to the nature of the UAS/GAL4 system, Xbp1-EGFP was likely to be over-expressed in tissues or cells, which may overload the mechanisms regulating Xbp1 mRNA targeting to the ER membrane. Hence, the question whether or not the HR2 and the CTR of Xbp1^unspliced^ are required for the targeting of Xbp1 mRNA to the ER membrane was not yet directly addressed in *Drosophila*. Here, we generated a deletion mutant (*Excision101*) of *Drosophila* Xbp1, lacking HR2 and CTR, but not the Ire1 splicing site. The transcription of Xbp1 mRNA in *Excision101* is still at normal levels, since the upstream regulatory regions were not deleted in this mutant. We found that Ire1-dependent splicing of Xbp1 mRNA is reduced in *Excision101*, but not completely abolished. Our results suggest the existence of additional mechanisms for ER membrane targeting of Xbp1 mRNA that are independent of the C-terminal domain of Xbp1^unspliced^.

## Methods

### *Drosophila* genetics and molecular biology

Flies and crosses were raised with standard cornmeal food, at 25°C. *Excision101* was generated by crossing the homozygous viable line carrying the transposon *P{SUPor-P}CG9418^KG05183^* with Δ23 transposase. Males with mosaic orange eyes were selected and crossed with double balancer females. White eyed male progeny were tested for lethality complementation with *P{lacW}xbp1* ^k13803^.

*Excision101* was balanced with *CyO-GFP* to collect homozygous mutant and heterozygote control larvae. Cages to collect larvae were set on apple juice plates and maintained at 25°C. For tunicamycin feeding experiments, 24 h. after egg laying larvae were exposed to yeast paste food with/without tunicamycin (5 µg/mL) for 6–8 hours and collected for RNA extraction.

Genomic DNA was isolated from larvae or flies using the High Pure PCR Template Preparation kit (Roche). Samples were snap frozen in liquid nitrogen and macerated with a motorized pestle in lysis buffer. The limits of *Excision101* were determined by PCR and sequencing with the primers 5′AGT GAC GTT GCT TGG CTG AGT GAC and 5′GCA GCA CAA CACCAG ATG C. Total RNA was extracted with *High Pure RNA Tissue* kit (Roche) and used to synthesize cDNA with random hexamers (RevertAid First Strand kit - Thermo/Fermentas). Quantitative reverse transcription-PCR (RT-PCR) analysis was performed on the cDNA obtained using SSoFast EvaGreen Supermix and a Bio-Rad Cfx-96 detection system. All samples were analyzed in triplicates and from 3 independent RNA extractions. For each sample, the levels of mRNAs were normalized using rp49 as a loading control. PCR primers were: rp49 (Fwd: 5′AGA TCG TGA AGA AGC GCA CCA AGC, Rev: 5′GCA CCA GGA ACTT CTT GAA TCC GG), Xbp1 (Fwd1: 5′CAT CAA CGA GTC ACT GCT GGC CAA G, Rev3: 5′CGC TGA CGA CTG TGT GTC C), Pdi (Fwd: 5′TCA TCG AGA GTC CTG TCC AGG TTG, Rev: 5′AAC ACC TCC TTT CCC AGG AGC TG) and BiP (Fwd: 5′TGT CAC CGA TCT GGT TCT TCA GGC, Rev: 5′GTC CCA TGA CCA AGG ACA ACC ATC).

### Analysis of Xbp1 mRNA splicing by PstI digestion and sequencing

Splicing of Xbp1 mRNA was accessed by restriction analysis with PstI of a fragment containing Ire1 splice site as described in [39]. *Excision101/CyO* heterozygous larvae were dissected in PBS and brains and eye imaginal discs were cultured in Schneider’s medium with or without 5 mM DTT for 5 hr. Total RNA was extracted with *High Pure RNA Tissue* kit (Roche) and used to synthesize cDNA with random hexamers (RevertAid First Strand kit - Thermo/Fermentas). Fragments flanking Ire1 splicing site were amplified using specific primers for the *CyO* chromosome (Fwd1 and Rev1) or *Excision101* (Fwd1 and Rev2). PCR amplification fragments were digested with PstI overnight. Primers sequence: Fwd1: 5′CAT CAA CGA GTC ACT GCT GGC CAAG, Rev1: 5′GTC TGC TGT GAT ATC TGC GAG CAG AC, Rev2: 5′CTG GTT AAT GCA GCT CTG CGA AGC C3’. For DNA sequencing, the *CyO* chromosome or *Excision101* specific PCR products were cloned in pJet (Thermo/Fermentas). Single colonies were grown for plasmid mini-preparation (NZYTech) and DNA sequencing was performed by Stab Vida using as sequencing primers Fwd1 and Rev1 or Rev2.

## Results and Discussion

### HR2 and CTR are conserved in *Drosophila* Xbp1^unspliced^

**Figure 2:**
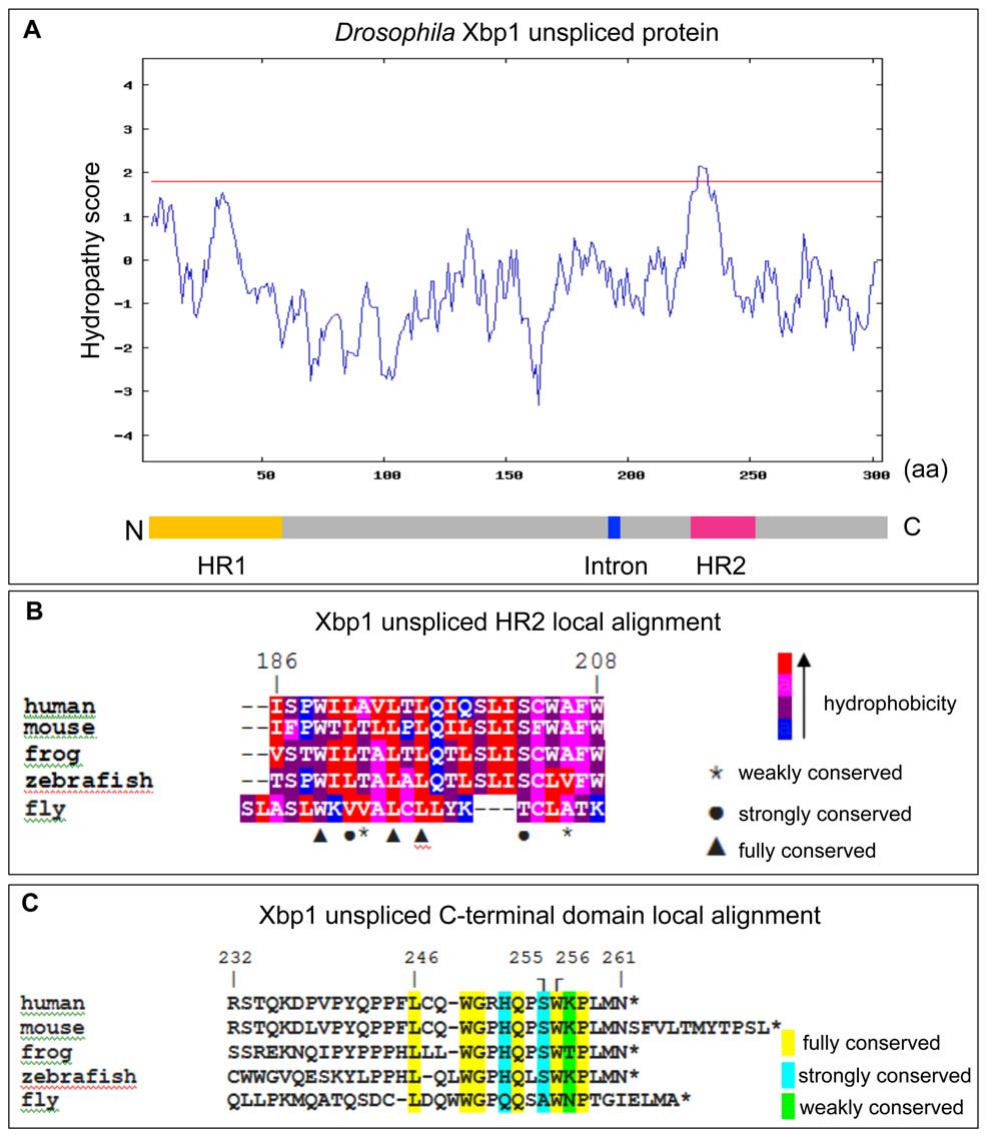
Conservation of HR2 and C-terminal region of Xbp1^unpsliced^. (A) Kyte and Doolittle hydrophobicity plot of *Drosophila* Xbp1^unspliced^, indicating the existence of two hydrophobic regions, HR1 and HR2. The horizontal red line indicates a score of 1.8. (B) Local amino acid sequence alignment of HR2 using several species. Amino acids that are fully, strongly or weakly conserved are indicated. The hydrophobicity of each amino acid is indicated by a color code. (C) Local amino acid sequence alignment of the C-terminal translational pausing region. Amino acids that are fully, strongly or weakly conserved are indicated.

The mechanism of activation of Xbp1 by Ire1-mediated unconventional splicing of Xbp1 mRNA exists in *Drosophila* [14], [15], [32], as well as RIDD, which was first described in *Drosophila* S2 cells [20]. Moreover, the main UPR components, including Ire1, ATF6 and Perk, have homologs in *Drosophila* with high conservation scores [40]. To elucidate if the mechanism of Xbp1 mRNA transport to the ER surface in *Drosophila* is conserved with mammals, we started by performing Kyte and Doolittle hydrophobicity analysis, which indicated the existence of a highly hydrophobic region in the carboxyl end of the *Drosophila* Xbp1^unspliced^ protein, corresponding to the HR2 (Figure 2A). Moreover, the *Drosophila* Xbp1^unspliced^ protein has, within HR2, several amino acid residues that are fully or strongly conserved with human and other vertebrate species (Figure 2B).

In humans, the L246A and W256A mutations in the C-terminal domain of Xbp1^unspliced^ abrogated translational pausing [31]. These Leucine and Tryptophan residues are conserved in humans, mice, zebrafish, *Xenopus* (frog) and *Drosophila,* further supporting the physiological role of these amino acid residues in translational pausing (Figure 2C). An S255-to-A255 mutation was previously reported to increase translational pausing in human Xbp1^unspliced^ [31]. Interestingly, in *Drosophila* this position is occupied by an Alanine residue instead of the Serine, which is found in all other species (Figure 2C), which suggests that translational pausing should occur in *Drosophila* Xbp1^unspliced^ with high efficiency.

### *Excision101* originates a truncated Xbp1 mRNA that is spliced by Ire1

**Figure 3:**
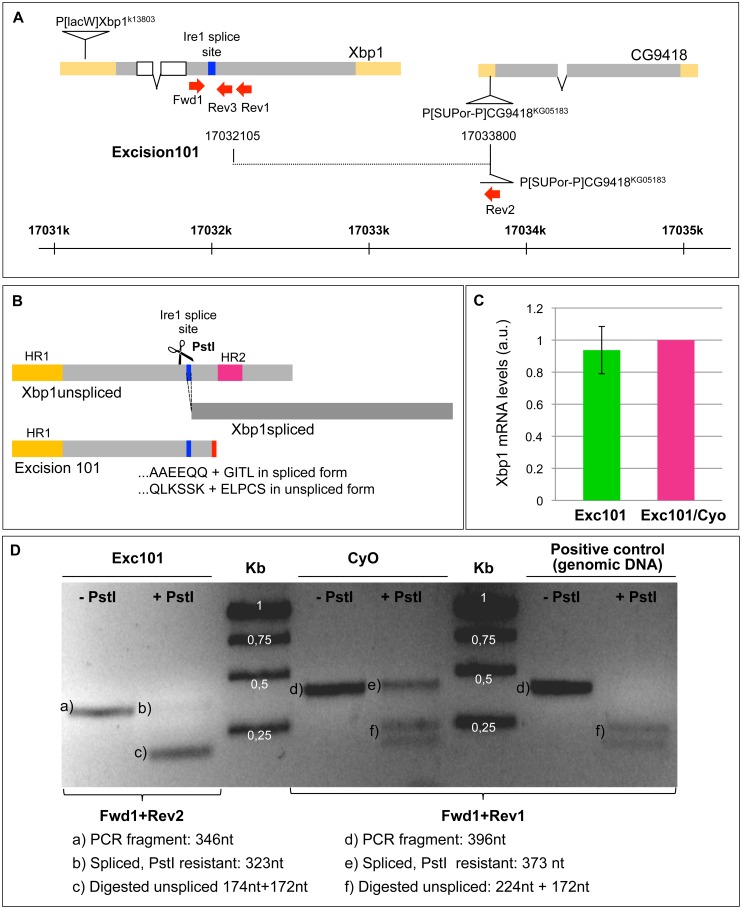
Excision101 originates a truncated Xbp1 mRNA that is spliced by Ire1. (A) Schematic representation of genomic region around Xbp1, with the localization of Excision101 deletion and the PCR primers Fwd1, Rev1, Rev2 and Rev3. In Excision 101, the breakpoint in *P{SUPor-P}CG9418^KG05183^* is 5′GAA TTA CCT TGT AGT TGA TAT TTG AGA T (following the leading strand of Xbp1). (B) Schematic representation of the Xbp1^unspliced^ and Xbp1^spliced^ proteins in “wild-type” and in *Excision101*. PstI has a cleavage site in the intron spliced by Ire1. HR1: hydrophobic region 1. HR2: hydrophobic region 2. (C) Quantitative RT-PCR for total Xbp1 mRNA levels in larvae homozygous for *Excision101*or heterozygous *Excision101/CyO-GFP*, using the primers for Xbp1, Fwd1 and Rev3. Control *Excision101/Cyo* is set as 1, with the homozygous *Excision101* Xbp1 mRNA levels indicated as a mean +/− standard deviation (a.u. - arbitrary units). (D) Agarose gel electrophoresis of RT-PCR products specific for *Excision101* or *CyO* control chromosome after digestion with PstI restriction enzyme. PCR product specific for the Xbp1^unspliced^ form is cleaved by PstI, while Xbp1^spliced^ form is resistant to PstI digestion (because the PstI is in the intron that is spliced by Ire1). A positive control using genomic DNA is fully digested by PstI.

We generated a mutant allele of Xbp1, *Excision101* (*Exc101*), by imprecise excision of *P{SUPor-P}CG9418^KG05183^*, a non-lethal transposable element inserted in the 5′UTR of *CG9418*, the gene immediately downstream of Xbp1. *Excision101* is a deletion that originates Xbp1 proteins with truncated C-terminal regions (Figure 3A). In the Xbp1^spliced^ open reading frame, the last 278 codons (at the 3′ region of the coding sequence) are deleted and 4 new codons (GITL) are present before the introduction of a nonsense STOP codon. In the Xbp1^unspliced^ frame, the last 87 codons are deleted and 5 new codons (ELPCS) are followed by a nonsense STOP codon (Figure 3B). We confirmed by quantitative RT-PCR that the transcriptional regulation of Xbp1 mRNA is not affected in *Excision101*. Larvae homozygous for *Excision101* present similar levels of Xbp1 mRNA to *Excision101/CyO-GFP* control larvae (Figure 3C).

Taking into account that the predicted Xbp1^unspliced^ protein encoded by *Excision101* does not have HR2 or CTR, we used two different assays to verify if *Excision101* mRNA is spliced by Ire1. In a first assay, we were able to distinguish the mRNA of the Xbp1^spliced^ form from the Xbp1^unspliced^ form by digestion with the restriction enzyme PstI of a cDNA/PCR-amplified fragment containing the Ire1 splicing site. A PstI restriction site is present in the Xbp1 intron that is removed by Ire1, and consequently PstI only cuts the fragment corresponding to the Xbp1^unspliced^ form. Heterozygous *Excision 101/Cyo* third instar larvae were dissected (cut in half) and treated with 5 mM DTT (dithiothreitol, in PBS or Schneider cells medium) for 5 hours, to activate Ire1. Total RNA was extracted and, after cDNA synthesis, PCR products were generated with primers that allow the specific amplification from *Excision 101* or the *CyO* (balancer chromosome that serves as “wild-type” control) alleles (Figure 3A, D). The *Excision101*-specific fragment was mostly digested with PstI, although a faint PstI-resistant band, corresponding to the Xbp1^spliced^, was also observed. So most of the *Excision101*-specific mRNA is not cleaved by Ire1 and remains in the Xbp1^unspliced^ form, even after 5 hours of DTT treatment. In the *CyO*-specific fragment, the PstI-resistant band (Xbp1^spliced^) is more prominent, indicating that more Xbp1 mRNA is spliced by Ire1 in this case. A positive control using genomic DNA was fully digested by PstI, indicating that the observed PstI-resistant fragments should not be due to incomplete digestion (Figure 3D).

**Figure 4:**
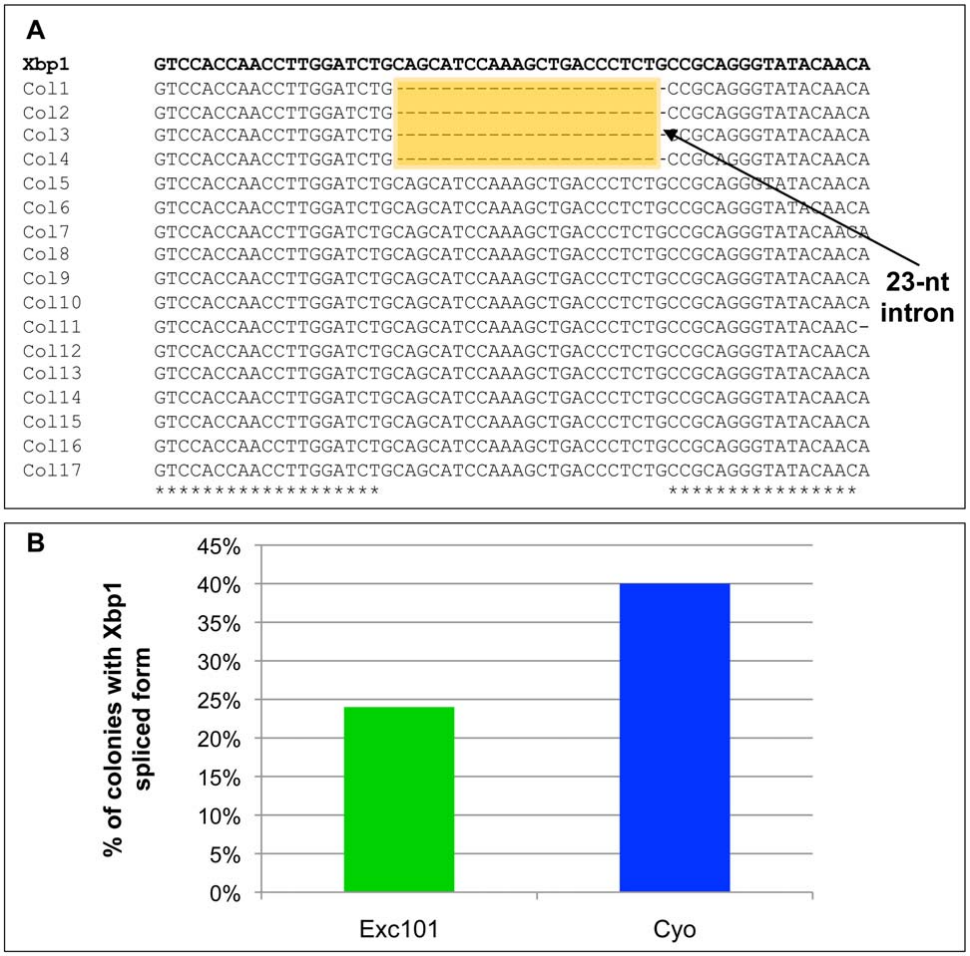
cDNA sequencing demonstrates splicing in *Excision101* specific mRNA. Sequencing of single colonies obtained after cloning of *Excision101* specific cDNA. The absence of the intron in colonies 1–4 demonstrates that *Excision101* mRNA is spliced by Ire1. Percentage of colonies containing Xbp1^spliced^ form in *Excision101* or *CyO* control chromosome. The total number of colonies sequenced was 38 for *Excision101* and 37 for the *CyO* control.

In a second approach, we sequenced plasmids from several single colonies that were obtained after cloning of the *Excision101* or *CyO* specific PCR fragments, to confirm whether or not the intron was present. In the *CyO* control, approximately 40% of the colonies (15 out of 37) corresponded to the Xbp1^spliced^ form (and 60% to the Xbp1^unspliced^ form). In *Excision101* approximately 23% of the colonies (9 out of 38) corresponded to the Xbp1^spliced^ form (and 77% to the Xbp1^unspliced^ form), which represents a reduction in the amount of Xbp1^spliced^ form, in comparison with the *CyO* control (Figure 4). These results indicate that the elements deleted from the 3′region of Xbp1 in *Excision101*, such as HR2 and CTR, are important for Ire1 mediated splicing of Xbp1 mRNA. Nevertheless, around 23% of the colonies obtained from *Excision101* amplification corresponded to the Xbp1^spliced^ form, which demonstrates that Xbp1 splicing still occurs, even in the absence of HR2 and translational pausing. It is possible that the HR1 of Xbp1^unspliced^, which is still present in *Excision101*, is sufficient to promote the association of Xbp1 mRNA with the ER membrane.

**Figure 5:**
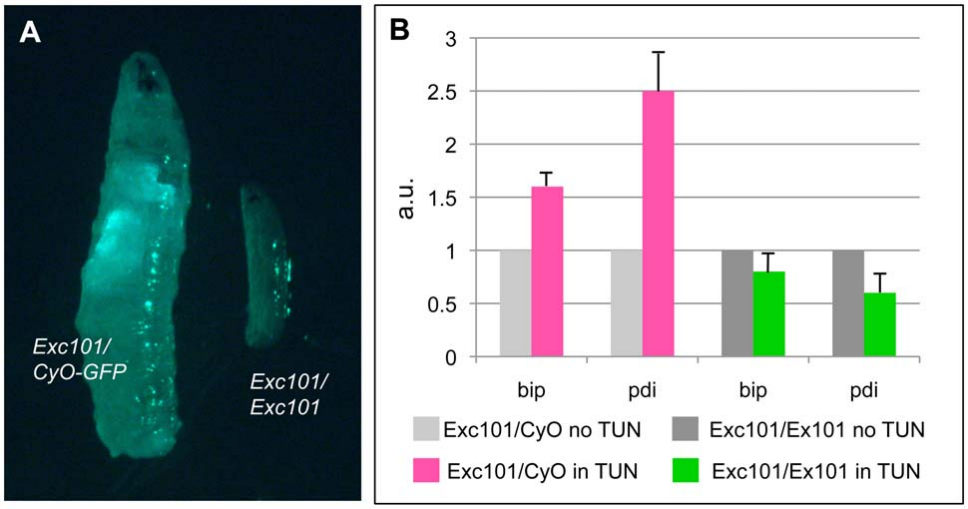
*Excision101* homozygous larvae have impaired UPR activation. (A) Picture of 3-day old *Excision101* homozygous larva and heterozygous *Excision101/CyO-GFP* sibling control. *Excision101* homozygous larva arrest development during first instar stage. (B) Quantitative RT-PCR for BiP and Pdi mRNA levels in *Excision101* homozygous or control heterozygous *Excision101/CyO-GFP* larvae treated with/without tunicamycin food. Induction of BiP and Pdi upon tunicamycin treatment is impaired in *Excision101* homozygous larva. mRNA levels are indicated as a mean +/− standard deviation (a.u. - arbitrary units).

Finally, we tested the susceptibility of *Excision101* homozygous larvae upon treatment with tunicamycin, an ER stress-inducing drug. Larvae homozygous for *Excision101* arrest development during the first instar stage and die 2 to 3 days after egg laying, while similarly aged heterozygous *Excision101/CyO-GFP* sibling control larvae develop normally (Figure 5A). In fact, lethality of *Excision101* homozygous larva occurs at the same stage than larva homozygous for *Excision 250*, a previously described total deletion of Xbp1 [24], suggesting that Xbp1^spliced^ from *Excision101* does retain little or no activity. This is expected, since the C-terminal half of Xbp1^spliced^ is deleted in *Excision101*. We exposed larva to yeast paste food containing tunicamycin and assayed for induction of ER stress markers (BiP and Pdi) by real time RT-PCR (Figure 5B). As expected, the induction of ER stress markers is compromised in organisms homozygous for *Excision 101*.

The features of C-terminal region in Xbp1^unspliced^ protein, including the high hydrophobicity profile and critical amino acid residues to translational pausing, are shared between vertebrates and invertebrates, suggesting that the mechanism to target Xbp1 mRNA to the ER membrane described in humans is conserved across metazoans. This terminal hydrophobic region 2 is predicted to form an alpha helix [30], which is important for the association with lipid bilayers, dragging the mRNA-ribosome-nascent polypeptide complex to the ER membrane. Using an Xbp1 mutant lacking the C-terminal region, we investigated the mechanism underlying the targeting of Xbp1 mRNA to the ER membrane in *Drosophila*. We found the lack of the C-terminal region decreases the splicing efficiency of Xbp1 mRNA, but it does not abolish splicing completely. Our results suggest the existence of additional mechanisms for ER membrane targeting of Xbp1 mRNA that are independent of the C-terminal domain of *Drosophila* Xbp1^unspliced^.

## References

[1] Moore KA, Hollien J (2012) The unfolded protein response in secretory cell function. Annu Rev Genet 46: 165–183. 22934644 10.1146/annurev-genet-110711-155644

[2] Ron D, Walter P (2007) Signal integration in the endoplasmic reticulum unfolded protein response. Nat Rev Mol Cell Biol 8: 519–529. 17565364 10.1038/nrm2199

[3] Walter P, Ron D (2011) The unfolded protein response: from stress pathway to homeostatic regulation. Science 334: 1081–1086. 22116877 10.1126/science.1209038

[4] Shamu CE, Walter P (1996) Oligomerization and phosphorylation of the Ire1p kinase during intracellular signaling from the endoplasmic reticulum to the nucleus. Embo J 15: 3028–3039. 8670804 PMC450244

[5] Tirasophon W, Welihinda AA, Kaufman RJ (1998) A stress response pathway from the endoplasmic reticulum to the nucleus requires a novel bifunctional protein kinase/endoribonuclease (Ire1p) in mammalian cells. Genes Dev 12: 1812–1824. 9637683 10.1101/gad.12.12.1812 PMC316900

[6] Liu CY, Wong HN, Schauerte JA, Kaufman RJ (2002) The protein kinase/endoribonuclease IRE1alpha that signals the unfolded protein response has a luminal N-terminal ligand-independent dimerization domain. J Biol Chem 277: 18346–18356. 11897784 10.1074/jbc.M112454200

[7] Credle JJ, Finer-Moore JS, Papa FR, Stroud RM, Walter P (2005) On the mechanism of sensing unfolded protein in the endoplasmic reticulum. Proc Natl Acad Sci U S A 102: 18773–18784. 16365312 10.1073/pnas.0509487102 PMC1316886

[8] Gardner BM, Walter P (2011) Unfolded proteins are Ire1-activating ligands that directly induce the unfolded protein response. Science 333: 1891–1894. 21852455 10.1126/science.1209126 PMC3202989

[9] Cox JS, Walter P (1996) A novel mechanism for regulating activity of a transcription factor that controls the unfolded protein response. Cell 87: 391–404. 8898193 10.1016/s0092-8674(00)81360-4

[10] Sidrauski C, Walter P (1997) The transmembrane kinase Ire1p is a site-specific endonuclease that initiates mRNA splicing in the unfolded protein response. Cell 90: 1031–1039. 9323131 10.1016/s0092-8674(00)80369-4

[11] Yoshida H, Matsui T, Yamamoto A, Okada T, Mori K (2001) XBP1 mRNA is induced by ATF6 and spliced by IRE1 in response to ER stress to produce a highly active transcription factor. Cell 107: 881–891. 11779464 10.1016/s0092-8674(01)00611-0

[12] Shen X, Ellis RE, Lee K, Liu CY, Yang K, et al (2001) Complementary signaling pathways regulate the unfolded protein response and are required for C. elegans development. Cell 107: 893–903. 11779465 10.1016/s0092-8674(01)00612-2

[13] Calfon M, Zeng H, Urano F, Till JH, Hubbard SR, et al (2002) IRE1 couples endoplasmic reticulum load to secretory capacity by processing the XBP-1 mRNA. Nature 415: 92–96. 11780124 10.1038/415092a

[14] Ryoo HD, Domingos PM, Kang MJ, Steller H (2007) Unfolded protein response in a Drosophila model for retinal degeneration. Embo J 26: 242–252. 17170705 10.1038/sj.emboj.7601477 PMC1782370

[15] Plongthongkum N, Kullawong N, Panyim S, Tirasophon W (2007) Ire1 regulated XBP1 mRNA splicing is essential for the unfolded protein response (UPR) in Drosophila melanogaster. Biochem Biophys Res Commun 354: 789–794. 17266933 10.1016/j.bbrc.2007.01.056

[16] Lee AH, Iwakoshi NN, Anderson KC, Glimcher LH (2003) Proteasome inhibitors disrupt the unfolded protein response in myeloma cells. Proc Natl Acad Sci U S A 100: 9946–9951. 12902539 10.1073/pnas.1334037100 PMC187896

[17] Lee AH, Scapa EF, Cohen DE, Glimcher LH (2008) Regulation of hepatic lipogenesis by the transcription factor XBP1. Science 320: 1492–1496. 18556558 10.1126/science.1158042 PMC3620093

[18] Yamamoto K, Sato T, Matsui T, Sato M, Okada T, et al (2007) Transcriptional induction of mammalian ER quality control proteins is mediated by single or combined action of ATF6alpha and XBP1. Dev Cell 13: 365–376. 17765680 10.1016/j.devcel.2007.07.018

[19] Yoshida H, Oku M, Suzuki M, Mori K (2006) pXBP1(U) encoded in XBP1 pre-mRNA negatively regulates unfolded protein response activator pXBP1(S) in mammalian ER stress response. J Cell Biol 172: 565–575. 16461360 10.1083/jcb.200508145 PMC2063676

[20] Hollien J, Weissman JS (2006) Decay of endoplasmic reticulum-localized mRNAs during the unfolded protein response. Science 313: 104–107. 16825573 10.1126/science.1129631

[21] Cross BC, Bond PJ, Sadowski PG, Jha BK, Zak J, et al (2012) The molecular basis for selective inhibition of unconventional mRNA splicing by an IRE1-binding small molecule. Proc Natl Acad Sci U S A 109: E869–878. 22315414 10.1073/pnas.1115623109 PMC3326519

[22] Kimmig P, Diaz M, Zheng J, Williams CC, Lang A, et al (2012) The unfolded protein response in fission yeast modulates stability of select mRNAs to maintain protein homeostasis. Elife 1: e00048. 23066505 10.7554/eLife.00048 PMC3470409

[23] Hollien J, Lin JH, Li H, Stevens N, Walter P, et al (2009) Regulated Ire1-dependent decay of messenger RNAs in mammalian cells. J Cell Biol 186: 323–331. 19651891 10.1083/jcb.200903014 PMC2728407

[24] Coelho DS, Domingos PM (2014) Physiological roles of regulated Ire1 dependent decay. Front Genet 5: 76. 24795742 10.3389/fgene.2014.00076 PMC3997004

[25] Maurel M, Chevet E, Tavernier J, Gerlo S (2014) Getting RIDD of RNA: IRE1 in cell fate regulation. Trends Biochem Sci 39: 245–254. 24657016 10.1016/j.tibs.2014.02.008

[26] Gaddam D, Stevens N, Hollien J (2013) Comparison of mRNA localization and regulation during endoplasmic reticulum stress in Drosophila cells. Mol Biol Cell 24: 14–20. 23135994 10.1091/mbc.E12-06-0491 PMC3530775

[27] Hollien J (2013) Evolution of the unfolded protein response. Biochim Biophys Acta 1833: 2458–2463. 23369734 10.1016/j.bbamcr.2013.01.016

[28] Moore KA, Plant JJ, Gaddam D, Craft J, Hollien J (2013) Regulation of sumo mRNA during endoplasmic reticulum stress. PLoS One 8: e75723. 24058701 10.1371/journal.pone.0075723 PMC3776770

[29] Aragon T, van Anken E, Pincus D, Serafimova IM, Korennykh AV, et al (2009) Messenger RNA targeting to endoplasmic reticulum stress signalling sites. Nature 457: 736–740. 19079237 10.1038/nature07641 PMC2768538

[30] Yanagitani K, Imagawa Y, Iwawaki T, Hosoda A, Saito M, et al (2009) Cotranslational targeting of XBP1 protein to the membrane promotes cytoplasmic splicing of its own mRNA. Mol Cell 34: 191–200. 19394296 10.1016/j.molcel.2009.02.033

[31] Yanagitani K, Kimata Y, Kadokura H, Kohno K (2011) Translational pausing ensures membrane targeting and cytoplasmic splicing of XBP1u mRNA. Science 331: 586–589. 21233347 10.1126/science.1197142

[32] Souid S, Lepesant JA, Yanicostas C (2007) The xbp-1 gene is essential for development in Drosophila. Dev Genes Evol 217: 159–167. 17206451 10.1007/s00427-006-0124-1

[33] Griciuc A, Aron L, Roux MJ, Klein R, Giangrande A, et al (2010) Inactivation of VCP/ter94 suppresses retinal pathology caused by misfolded rhodopsin in Drosophila. PLoS Genet 6. 10.1371/journal.pgen.1001075 PMC2928793 20865169

[34] Kang MJ, Ryoo HD (2009) Suppression of retinal degeneration in Drosophila by stimulation of ER-associated degradation. Proc Natl Acad Sci U S A 106: 17043–17048. 19805114 10.1073/pnas.0905566106 PMC2749843

[35] Mendes CS, Levet C, Chatelain G, Dourlen P, Fouillet A, et al (2009) ER stress protects from retinal degeneration. Embo J 28: 1296–1307. 19339992 10.1038/emboj.2009.76 PMC2683051

[36] Coelho DS, Cairrao F, Zeng X, Pires E, Coelho AV, et al (2013) Xbp1-independent Ire1 signaling is required for photoreceptor differentiation and rhabdomere morphogenesis in Drosophila. Cell Rep 5: 791–801. 24183663 10.1016/j.celrep.2013.09.046 PMC3858604

[37] Brand AH, Perrimon N (1993) Targeted gene expression as a means of altering cell fates and generating dominant phenotypes. Development 118: 401–415. 8223268 10.1242/dev.118.2.401

[38] Sone M, Zeng X, Larese J, Ryoo HD (2012) A modified UPR stress sensing system reveals a novel tissue distribution of IRE1/XBP1 activity during normal Drosophila development. Cell Stress Chaperones 18: 307–319. 23160805 10.1007/s12192-012-0383-x PMC3631089

[39] Casas-Tinto S, Zhang Y, Sanchez-Garcia J, Gomez-Velazquez M, Rincon-Limas DE, et al (2011) The ER stress factor XBP1s prevents amyloid-beta neurotoxicity. Hum Mol Genet 20: 2144–2160. 21389082 10.1093/hmg/ddr100 PMC3090193

[40] Ryoo HD, Steller H (2007) Unfolded protein response in Drosophila: why another model can make it fly. Cell Cycle 6: 830–835. 17387279 10.4161/cc.6.7.4064

